# Hypoglycaemia incidence and recovery during home use of hybrid closed‐loop insulin delivery in adults with type 1 diabetes

**DOI:** 10.1111/dom.13304

**Published:** 2018-04-16

**Authors:** Yue Ruan, Lia Bally, Hood Thabit, Lalantha Leelarathna, Sara Hartnell, Martin Tauschmann, Malgorzata E. Wilinska, Mark L. Evans, Julia K. Mader, Harald Kojzar, Sibylle Dellweg, Carsten Benesch, Sabine Arnolds, Thomas R. Pieber, Roman Hovorka

**Affiliations:** ^1^ Wellcome Trust–MRC Institute of Metabolic Science University of Cambridge Cambridge UK; ^2^ Department of Diabetes, Endocrinology, Clinical Nutrition and Metabolism Inselspital, Bern University Hospital, University of Bern Bern Switzerland; ^3^ Department of General Internal Medicine, Inselspital Bern University Hospital, University of Bern Bern Switzerland; ^4^ Central Manchester University Hospitals NHS Foundation Manchester Academic Health Science Centre Manchester UK; ^5^ Division of Diabetes, Endocrinology and Gastroenterology, Faculty of Biology, Medicine and Health University of Manchester Manchester UK; ^6^ Department of Diabetes and Endocrinology Cambridge University Hospitals NHS Foundation Trust Cambridge UK; ^7^ Department of Paediatrics University of Cambridge Cambridge UK; ^8^ Department of Internal Medicine, Division of Endocrinology & Diabetology Medical University of Graz Graz Austria; ^9^ Profil Institut fuer Stoffwechselforschung GmbH Neuss Germany

**Keywords:** continuous glucose monitoring (CGM), CSII, glycaemic control, hypoglycaemia, insulin delivery, type 1 diabetes

## Abstract

Glucose excursion was assessed prior to and post hypoglycaemia to increase understanding of hypoglycaemia incidence and recovery during hybrid closed‐loop insulin delivery. We retrospectively analysed data from 60 adults with type 1 diabetes who received, in a crossover randomized design, day‐and‐night hybrid closed‐loop insulin delivery and insulin pump therapy, the latter with or without real‐time continuous glucose monitoring. Over 4‐week study periods, we identified hypoglycaemic episodes, defined as sensor glucose <3.0 mmol/L, and analysed sensor glucose relative to the onset of hypoglycaemia. We identified 377 hypoglycaemic episodes during hybrid closed‐loop intervention vs 662 during control intervention (*P* < .001), with a predominant reduction of nocturnal hypoglycaemia. The slope of sensor glucose prior to hypoglycaemia was steeper during closed‐loop intervention than during control intervention (*P* < .01), while insulin delivery was reduced (*P* < .01). During both day and night, participants recovered from hypoglycaemia faster when treated by closed‐loop intervention. At 120 minutes post hypoglycaemia, sensor glucose levels were higher during closed‐loop intervention compared to the control period (*P* < .05).

In conclusion, closed‐loop intervention reduces the risk of hypoglycaemia, particularly overnight, with swift recovery from hypoglycaemia leading to higher 2‐hour post‐hypoglycaemia glucose levels.

## 1 INTRODUCTION

In type 1 diabetes, hypoglycaemia is a major barrier to achieving euglycaemia using modern tight glycaemic control strategies.^1^ It may be accompanied by sweating, trembling and confusion, and may require assistance from another person.^2^

Insulin pump therapy has been shown to reduce glycated haemoglobin levels without increasing the risk of hypoglycaemia.^3^ Continuous glucose monitoring also leads to improved glucose control and reduces the risk of hypoglycaemia^4^ and severe hypoglycaemia in adults with type 1 diabetes.^5^ Closed‐loop glucose control, combining insulin delivery with real‐time glucose sensing to administer insulin in a glucose‐responsive fashion, further improves glucose control.^6^ However, hypoglycaemia continues to be of concern during closed‐loop insulin delivery. Detailed assessments of hypoglycaemia timing, incidence and other characteristics during home use of closed‐loop insulin delivery are undocumented.

In the present analysis, we retrospectively assessed hypoglycaemic episodes from a large dataset comprising sensor glucose and insulin delivery involving 60 adults with type 1 diabetes who participated in a randomized crossover study contrasting day‐and‐night hybrid closed‐loop insulin delivery and sensor‐augmented or conventional pump therapy. We report data over 4‐week intervention periods and describe diurnal distribution of hypoglycaemia events and describe glucose excursion and insulin delivery before, during and after hypoglycaemic episodes.

## 2 METHODS

### 2.1 Experimental data

We retrospectively analysed 4‐week‐long periods of sensor glucose and insulin delivery data collected in 60 adults (27 from Cambridge, UK; 22 from Graz, Austria; 11 from Profil, Germany) with type 1 diabetes (31 male; mean age, 40.0 [11.2] years; mean BMI, 25.2 [3.8] kg/cm^2^; baseline HbA1c, 7.7 [0.9]%; duration of diabetes, 22.1 [10.4] years; total daily insulin, 0.57 [0.14] U/kg).^7^, ^8^

Participants were randomly assigned to receive, in a crossover randomized fashion, hybrid day‐and‐night closed‐loop insulin delivery and sensor‐augmented (32 participants) or conventional (28 participants) pump therapy. The participants' pre‐study rapid‐acting insulin analogue (aspart or lispro) was used during the study. Real‐time (closed‐loop and sensor‐augmented pump therapy) or masked (conventional pump therapy) glucose levels were measured using a continuous glucose monitoring device (FreeStyle Navigator II, Abbott Diabetes Care, Alameda, California) calibrated according to the manufacturer's instructions. The built‐in bolus wizard of the study insulin pump (Dana Diabecare R, SOOIL, Seoul, Republic of Korea) was used by participants to calculate insulin boluses at mealtimes and when administering correction boluses. During the closed‐loop period, a model‐predictive control algorithm directed basal insulin delivery.^7^, ^8^

A hypoglycaemic episode was defined as sensor glucose <3 mmol/L for at least 10 minutes.^9^ Hypoglycaemic episodes that were at least 30 minutes apart were counted as separate events. We excluded episodes within 60 minutes of insulin bolus as these episodes may be predominantly attributable to bolus over‐delivery and unrelated to closed‐loop glucose control. The exclusion criterion was applied to both study periods.

### 2.2 Statistical analysis

We identified hypoglycaemic episodes for each participant separately. We evaluated for each participant the average sensor glucose and the average basal insulin infusion rates from −60 to 120 minutes in 10‐minute steps relative to the onset of hypoglycaemic episodes. We then calculated the mean sensor glucose excursions and mean basal insulin infusion across all participants. The minimum glucose levels during hypoglycaemia, the area‐under‐curve (AUC) hypoglycaemia and the duration of hypoglycaemia were also calculated. Hypoglycaemic episodes identified during the night (midnight to 6:00 am) and during the day (6:00 am to midnight) were analysed separately.

A Student's t‐test contrasted endpoints collected during closed‐loop and control periods. Statistical analyses were performed using SPSS, version 21 (IBM Software, Hampshire, UK). *P* values less than .05 were considered statistically significant. Data are presented as mean (SD) unless stated otherwise.

## 3 RESULTS

Data from 1680 days of closed‐loop insulin delivery and 1680 days of sensor‐augmented or conventional insulin pump therapy were analysed. We identified 377 hypoglycaemic episodes during the closed‐loop period, of which 87 were nocturnal (midnight to 6:00 am), as compared to 662 episodes during the control period, of which 205 were nocturnal (closed‐loop vs control arm, 1.27 [1.17] vs 2.48 [2.50] episodes per participant per week; *P* < .001).

**Figure 1:**
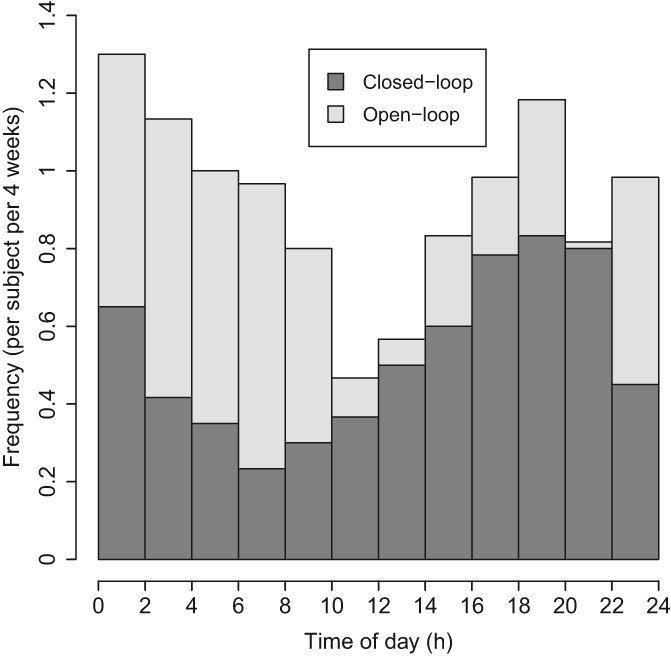
Incidence of hypoglycaemia events (sensor glucose <3.0 mmol/L for at least 10 minutes) during hybrid closed‐loop insulin delivery (dark grey bars) and control periods (light grey bars) (mean; N = 60)

Figure 1 shows the diurnal distribution of hypoglycaemia incidence during 2 treatment periods. A reduced incidence of hypoglycaemia was observed during the closed‐loop period as compared to the control period, with a predominant reduction between 10:00 pm and 8:00 am when the incidence of hypoglycaemia was halved. Figure S1 shows the risk of hypoglycaemia conditioned on ambient sensor glucose; with sensor glucose between 3 and 8 mmol/L, the risk of hypoglycaemia 60 minutes later is halved during closed‐loop intervention.

**Figure 2:**
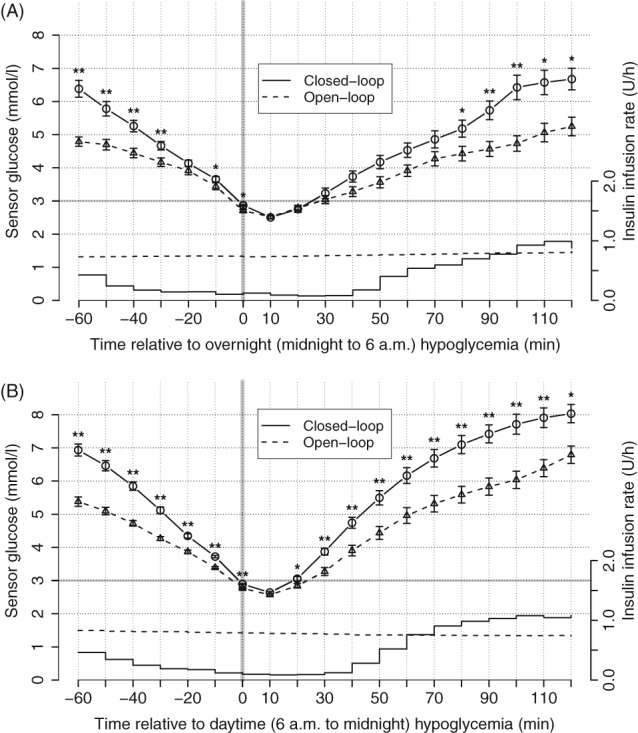
Sensor glucose values from −60 to 120 minutes relative to the onset of hypoglycaemia (sensor glucose <3.0 mmol/L; vertical bar) during the hybrid closed‐loop period (circles connected by solid line; mean ± SEM; N = 60; ^*^ *P* < .05, ^**^ *P* < .01 compared to control therapy) and during the control period (triangles connected by dashed line). Piecewise‐constant lines without error bars represent mean insulin infusion rates during the closed‐loop period and dashed lines without error bars represent insulin infusions during the control period. Panel A, shows glycaemic and insulin infusion data during the night period (midnight to 6:00 am) and panel B, shows the day period (6:00 am to midnight)

Figure 2 summarizes sensor glucose levels before, during and after hypoglycaemic episodes during closed‐loop and control periods. Sensor glucose prior to hypoglycaemia had a steeper decline during closed‐loop intervention as compared to the control period (*P* = .002). During the day, participants recovered from hypoglycaemia more rapidly when treated by closed‐loop intervention (higher sensor glucose values from 20 to 120 minutes post hypoglycaemia; *P* < .05). A similar trend was observed during the night. Table S1 shows sensor glucose values from 30 to 120 minutes relative to the onset of hypoglycaemia.

Mean basal insulin infusion rates were lower during closed‐loop intervention as compared to control periods, from −60 to 80 minutes during the day (*P* = .001) and from −60 to 50 minutes during the day (*P* = .003), respectively.

Minimum glucose levels during hypoglycaemia did not differ between closed‐loop and open‐loop intervention (2.4 [0.4] vs 2.5 [0.4] mmol/L; *P* = .4 for the night period and 2.6 [0.2] vs 2.5 [0.4] mmol/L; *P* = .1 for the day period). AUC hypoglycaemia was reduced during closed‐loop compared to open‐loop intervention (40.3 [33.1] vs 52.8 [43.9] mmol/L min; *P* = .04 for the night period and 22.4 [8.8] vs 38.8 [52.9] mmol/L min; *P* = .02 for the day period). The duration of hypoglycaemia was reduced by 21 minutes during the closed‐loop night period (51.9 [30.3] vs 72.9 [37.8] minutes; *P* < .001), with no difference during the day (35.2 [11.9] vs 45.5 [24.4] minutes; *P* = .06].

## 4 DISCUSSION

The present analysis reports the incidence and diurnal distribution of hypoglycaemia in adults with type 1 diabetes during home use of hybrid closed‐loop insulin delivery and sensor‐augmented or conventional insulin pump therapy. We evaluated sensor glucose excursions and basal insulin infusion rates prior to and post hypoglycaemia. We found different patterns of hypoglycaemia incidence and hypoglycaemia recovery between the 2 interventions.

Many prospective and retrospective studies of hypoglycaemia incidence are based on self‐reported data, with considerable variation in reported outcomes (eg, 43 episodes per patient‐year,^10^ 73 episodes per patient‐year^11^ and 94 episodes per patient‐year^12^). In the present analysis, we report the incidence of clinically significant hypoglycaemia at 144 episodes per patient‐year during insulin pump therapy using sensor glucose data. Continuous glucose monitoring provides comprehensive glucose levels over 24 hours and enables transparent definition and recording of hypoglycaemic episodes when device usage is high, as in the present analysis with median sensor wear time at 94% and 95% of the total time for closed‐loop and control periods, respectively.

Our analyses document the risk of hypoglycaemia being reduced during closed‐loop compared to control periods (377 vs 662 hypoglycaemic episodes) in adults with type 1 diabetes and with baseline HbA1c levels ranging from 5.8% to 9.7%. Figure 1 shows that the predominant reduction in hypoglycaemia was during the night. Figure 2 shows that, during closed‐loop periods, sensor glucose reduced more rapidly prior to hypoglycaemia as compared to control intervention. Our interpretation is that closed‐loop intervention was capable of preventing hypoglycaemia when sensor glucose was not decreasing rapidly. Thus, only a rapid decline in sensor glucose leads to hypoglycaemia during closed‐loop intervention. This is supported by reducing the risk of hypoglycaemia within 60 minutes, stratified according to ambient sensor glucose (Figure S1).

Two peaks of hypoglycaemia incidence were observed during 2 treatment periods, 1 at approximately 4:00–8:00 pm and the other at approximately 12:00–2:00 am (Figure 1). The former may be related to increased physical activity and the latter may result from post‐meal insulin corrections because of delayed effects following high‐fat evening meals.^13^

Previous studies have shown that closed‐loop intervention improves glycaemic control in type 1 diabetes through the system's ability to adjust insulin delivery in response to varying insulin requirements.^14^ Figure 2 demonstrates this paradigm; comparing the mean insulin delivery at approximately 0.8 U/h observed even during imminent onset of hypoglycaemia, closed‐loop intervention reduced insulin delivery from −60 to 80 minutes relative to onset of overnight hypoglycaemia and from −60 to 50 minutes relative to daytime hypoglycaemia. The reduced amount of insulin resulted in more rapid recovery and higher 2‐hour post‐hypoglycaemia glucose levels during closed‐loop intervention (Table S1 and Figure 2).

Glucose troughs during hypoglycaemia did not differ during closed‐loop and open‐loop interventions. However, during closed‐loop intervention, both AUC hypoglycaemia and the duration of hypoglycaemic events were reduced because of a more rapid recovery from hypoglycaemia.

An observational study reported that, in real‐life settings, a majority of patients over treated their hypoglycaemic episodes.^15^ Given that post‐hypoglycaemia glucose levels were higher during closed‐loop intervention as compared to control periods, a reasonable recommendation for clinical practitioners would be to reinforce, and possibly revise, patient education concerning hypoglycaemia correction, especially for those undergoing closed‐loop treatment. Further studies are warranted to explore optimal strategies for hypoglycaemia treatment during closed‐loop glucose control.

The strengths of our analysis are the multicentre, multinational, crossover, randomized study design, in which each subject serves as his/her own control, and the considerable volume of sensor glucose data used to identify the hypoglycaemic episodes. The data were collected during unsupervised home studies and, thus, glucose excursions reflect hypoglycaemia incidence and self‐treatment of hypoglycaemia under free‐living settings. The limitations include the lack of reliable data concerning the amount of rescue carbohydrates.

In conclusion, hybrid closed‐loop intervention reduces the risk of hypoglycaemia, particularly during the night, with a swift recovery from hypoglycaemia during the day, and leads to slightly elevated 2‐hour post‐hypoglycaemia glucose levels compared to those with insulin pump therapy.

## Supporting information


**Figure S1.** Probability of observing a sensor glucose value <3 mmol/L (y‐axis) conditional on the sensor glucose value 60 minutes earlier (x‐axis).
**Table S1.** Sensor glucose values at 30, 60, 90 and 120 min following the onset of hypoglycaemia (sensor glucose <3.0 mmol/L for at least 10 min) during hybrid closed‐loop insulin delivery and control periods.
