# Supplementary material for: Hypoglycaemia incidence and recovery during home use of hybrid closed‐loop insulin delivery in adults with type 1 diabetes
Source: Diabetes Obes Metab. 2018 Apr 16;20(8):2004–8. doi: 10.1111/dom.13304 (PMC6043360; doi:10.1111/dom.13304)
Supplement: Supplementary file 1 — Figure S1. Probability of observing a sensor glucose value <3 mmol/L (y‐axis) conditional on the sensor glucose value 60 minutes earlier (x‐axis). Table S1. Sensor glucose values at 30, 60, 90 and 120 min following the onset of hypoglycaemia (sensor glucose <3.0 mmol/L for at least 10 min) during hybrid closed‐loop insulin delivery and control periods. [file DOM-20-2004-s001.docx]

**Table S1. Sensor glucose values at 30, 60, 90 and 120 min following the onset of hypoglycaemia (sensor glucose < 3.0 mmol/l for at least 10 min) during hybrid closed-loop insulin delivery and control periods.**

| Time relative to onset of hypoglycaemia (min) | Sensor glucose (mmol/l)  [mean(SD), N = 60] | | P value |
| --- | --- | --- | --- |
|  | Control | Closed-loop insulin delivery |  |
|  | Overnight period (midnight to 6 a.m.) | |  |
| +30 | 3.0 (0.9) | 3.2 (1.2) | 0.80 |
| +60 | 3.9 (1.3) | 4.5 (1.7) | 0.53 |
| +90 | 4.6 (1.8) | 5.7 (2.2) | 0.003 |
| +120 | 5.2 (2.2) | 6.7 (2.5) | 0.010 |
|  | Daytime period (6 a.m. to midnight) | |  |
| +30 | 3.3 (0.9) | 3.9 (0.8) | 0.006 |
| +60 | 5.0 (1.8) | 6.2 (1.8) | 0.007 |
| +90 | 5.8 (2.0) | 7.4 (2.1) | 0.001 |
| +120 | 6.8 (2.0) | 8.0 (2.1) | 0.020 |


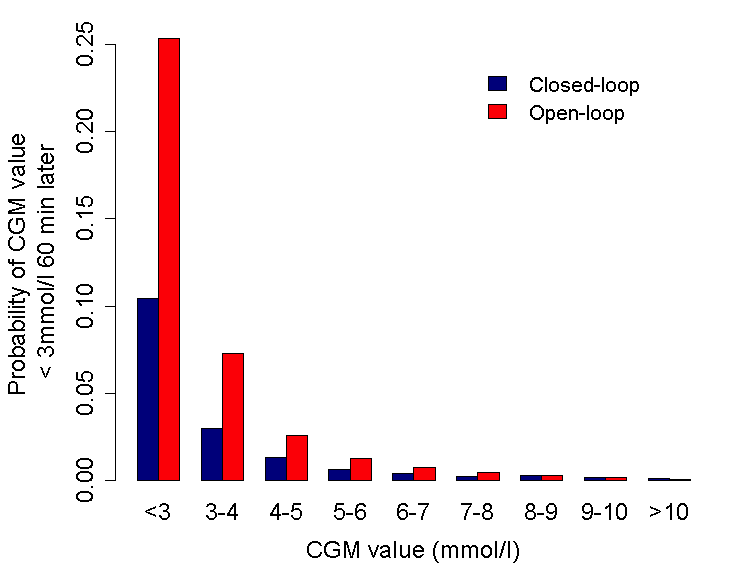


**Figure S1. Probability of observing a sensor glucose value < 3mmol/l (y-axis) conditional on the sensor glucose value 60 min earlier (x-axis).**
